# Advances in sequencing and omics studies in prostate cancer: unveiling molecular pathogenesis and clinical applications

**DOI:** 10.3389/fonc.2024.1355551

**Published:** 2024-05-10

**Authors:** Bingnan Lu, Yifan Liu, Yuntao Yao, Tianyue Yang, Haoyu Zhang, Xinyue Yang, Runzhi Huang, Wang Zhou, Xiuwu Pan, Xingang Cui

**Affiliations:** ^1^ Department of Urology, Xinhua Hospital Affiliated to Shanghai Jiao Tong University School of Medicine, Shanghai, China; ^2^ Shanghai Jiao Tong University School of Medicine, Shanghai, China; ^3^ Department of Burn Surgery, The First Affiliated Hospital of Naval Medical University, Shanghai, China

**Keywords:** prostate cancer, sequencing, omics, molecular pathogenesis, clinical application

## Abstract

**Background:**

Prostate cancer (PCa) is one of the most threatening health problems for the elderly males. However, our understanding of the disease has been limited by the research technology for a long time. Recently, the maturity of sequencing technology and omics studies has been accelerating the studies of PCa, establishing themselves as an essential impetus in this field.

**Methods:**

We assessed Web of Science (WoS) database for publications of sequencing and omics studies in PCa on July 3rd, 2023. Bibliometrix was used to conduct ulterior bibliometric analysis of countries/affiliations, authors, sources, publications, and keywords. Subsequently, purposeful large amounts of literature reading were proceeded to analyze research hotspots in this field.

**Results:**

3325 publications were included in the study. Research associated with sequencing and omics studies in PCa had shown an obvious increase recently. The USA and China were the most productive countries, and harbored close collaboration. CHINNAIYAN AM was identified as the most influential author, and CANCER RESEARCH exhibited huge impact in this field. Highly cited publications and their co-citation relationships were used to filtrate literatures for subsequent literature reading. Based on keyword analysis and large amounts of literature reading, ‘the molecular pathogenesis of PCa’ and ‘the clinical application of sequencing and omics studies in PCa’ were summarized as two research hotspots in the field.

**Conclusion:**

Sequencing technology had a deep impact on the studies of PCa. Sequencing and omics studies in PCa helped researchers reveal the molecular pathogenesis, and provided new possibilities for the clinical practice of PCa.

## Graphical Abstract

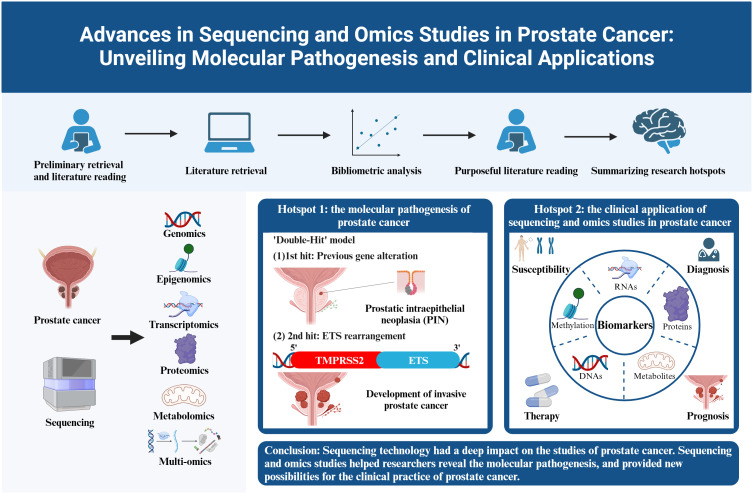

## 1 Introduction

Prostate cancer (PCa), is estimated to become the second most common cancer in males, accounting for 14.1% of all cancer cases according to the global cancer statistics of 2020 (1). Besides, the specific mortalities attributed to PCa reached 375,304 worldwide in 2020, ranking fifth in all cancers (1). PCa is still a threatening health problem in the face of elderly males. Nowadays, the diagnosis of PCa mainly relied on physical examination, prostate-specific antigen (PSA) screening, and prostate biopsy. As for the treatments of PCa, androgen deprivation therapy (ADT) as well as prostatectomy were the most common strategies for localized PCa. However, for non-metastatic castration resistant prostate cancer (nmCRPC) and metastatic castration resistant prostate cancer (mCRPC), some novel drugs including androgen pathway inhibitors (APIs) and inhibitors of Poly ADP Ribose Polymerase (PARP1) (PARPi) had shown their unique therapeutic effects in PCa (2). The challenges in the field of PCa mainly existed in the lack of understanding of its molecular pathogenesis, the short of effective biomarkers for the early diagnosis and prognosis, and the resistance to the current therapies. But for many years, our grasp of PCa was hindered by the limitation of technology and the biological heterogeneity of PCa. Nevertheless, in the past few decades, with the development of sequencing technology and the occurrence of corresponding omics studies, the research on PCa has gained a huge leap. A more comprehensive landscape of PCa is gradually coming into our view.

Sequencing technology refers to the technology detecting the sequence of biomacromolecules in the central dogma of molecular biology, which covers determining base sequences in a DNA or RNA molecule, and the order of amino acids in a protein. In 1977, Sanger sequencing was invented by Walter Gilbert and Frederick Sanger, and scientists were able to detect the sequence of nucleic acid for the first time (3). However, the length of Sanger sequencing was restricted to 700-1000 bp, which obviously could not satisfy the urgent demand of gene sequences in modern biology. With the completion of ‘human gene project’ in 2003, the sequencing technology was propelled onto an accelerated trajectory of development (4). Around 2005, high-throughput sequencing, also called next-generation sequencing (NGS), broke the restriction of measuring one sequence at one time, and could detect millions of sequences in a single run (5, 6). The giant breakthrough in the field of DNA sequencing consequently catalyzed an unprecedented surge in genomics studies. In the subsequent period, with the maturity of RNA sequencing and protein sequencing, transcriptomics and proteomics had gained remarkable advancement (7, 8). Notably, the first single-cell RNA sequencing method (scRNA-seq) was developed in 2009, and brought a huge leap for the field of sequencing. ScRNA-seq could detect the heterogeneity of each single cell, and analyze the biological process at a microscopic resolution, providing solutions to many puzzled problems in the past (9). More recently, in 2011, single-molecule sequencing was developed, overcoming the disadvantage of NGS in the necessary preliminary PCR process and could directly sequence a single molecule without prior amplification step, which took sequencing technology into the third revolution (10).

The development of sequencing technology and the prosperity of the corresponding omics studies have achieved great success in PCa, especially for the discovery of molecular pathogenesis, and the identification of biomarkers or targets for the susceptibility, diagnosis, therapy, and prognosis of PCa. The genomics and epigenomics studies identified many important genetic alterations, which provided new insights into the possible pathogenesis of PCa. RNA-seq, particularly scRNA-seq, further detected the transcriptional profile of PCa, and identified a series of differentially expressed genes (DEGs) between cancer tissues and normal tissues. Besides, the application of mass spectrometry (MS) had unveiled a repertoire of crucial proteins and metabolites. These biomacromolecules held immense potential as prognostic and diagnostic predictors, or as prospective therapeutic targets of PCa.

Bibliometrics refers to the interdisciplinary science of quantitative analysis of all knowledge carriers by mathematical and statistical methods, which originated in the early 20th century and became an independent discipline in 1969. Bibliometrics analyzes published information (such as journal articles, books) and its associated metadata (such as citations, abstracts, and keywords), in order to characterize or demonstrate correlations between published works (11). A number of bibliometric studies have been conducted in the field of rheumatism, spinal cord injury, COVID-19, etc. (12–14). However, there has not been a comprehensive bibliometric study concerned with sequencing and omics studies in PCa. After a preliminary retrieval and reading several associated literatures, we discerned the vital significance of sequencing and omics studies towards PCa, and obtained large amounts of related literatures. Thus, we intended to conduct an objective and quantitative analysis of these literatures using the methods of bibliometrics, and proceed purposeful literature reading on the basis of this, in order to comprehensively understand the historical trajectory and research hotspots in this field.

## 2 Materials and methods

### 2.1 Data sources and retrieval strategies

The WoS database includes over 13,000 of the world’s most influential academic journals, spanning various fields including natural sciences, engineering, biomedical sciences, etc. Besides, the WoS is the only database that could export full record and cited references, which is essential for bibliometric analysis. Thus, we chose the WoS database to conduct the bibliometric study. Our search term was as follows: ((TS=((prostate OR prostatic) NEAR/2 (cancer* OR tumor* OR tumor* OR cancer* OR carcinoma* OR adenocarcinoma OR oncology))) AND ((TI=transcriptomic) OR (TI=proteome) OR (TI=proteomics) OR (TI=metabolomics) OR (TI=bioinformatics) OR (TI=metagenomics) OR (TI=metatranscriptomics) OR (TI=omics) OR (TI=microarray) OR (TI=sequence) OR (TI=RNA-seq) OR (TI=sequencing) OR (TI=ATAC-seq) OR (TI=single cell sequencing) OR (TI= single cell sequence) OR (TI=single cell RNA sequencing) OR (TI= single cell RNA sequence) OR (TI= expression profile) OR (TI= bioinformatic*) OR (TI= high throughput))). Up to July 3rd, 2023, a total of 7743 publications were retrieved. Selecting research articles and reviews, only 3499 publications were left. Finally, we filtrated 3325 publications associated in the WoS database (core collection) for further analysis.

### 2.2 Bibliometric methodology

Full records and cited references of the 3325 publications retrieved were downloaded from WoS, and put into further analysis. Bibliometrix is an R package developed by Massimo Aria and Corrado Cuccurullo based on bibliometrics. Through the visualization of the retrieved results using Bibliometrix, we could quickly understand the classic publications and leading figures in the field, and analyze the futural developing trend as well (15). VOSviewer is a computer program for bibliometric mapping based on the data normalization approach of probability theory, and is capable of generating a visualized map of collaborative network analysis, keyword co-occurrence analysis, co-citation analysis, coupling analysis and so on (16). Citespace is a practical visualized analysis tool for bibliometric research developed by Professor Chaomei Chen, which uses the data normalization approach of aggregation theory to accomplish the similarity evaluation of knowledge units (17). VOSviewer and Citespace were used as supplementary analysis tools for our study.

Quantitative statistics were made by Bibliometrix. In the source analysis and author analysis, H index, G index, and M index are used to evaluate the local impact of certain source or author. H index is defined as the number of documents with citation number >= H, which is used to characterize a journal’s or scholar’s scientific output and local impact (18). G index is defined as follows: given a set of articles ranked in decreasing order of citations, the G index is the (unique) largest number such that the top G articles receive (together) at least g^2^ citations (19). The M index is an author’s H index/the years since the first publication (20). Besides, local citations refer to the citations in the retrieving field, while total citations refer to the citations in the global field, and the above two indexes could be used to evaluate the local and total impact, respectively. Bradford Law is used to describe the constructure of literatures in a field. The specific content is as follows: if journals are arranged in a descending order according to publications in a certain field, we could distinguish these journals into several parts with publications’ ratio as 1: α: α^2^: ⋯ (α > 1). The first part is the core area with the highest outputs (21). Lotka’s Law is stated as follows: the number of authors publishing n articles approximately accounts for 1/n^2^ of the number of authors publishing one article in a certain field (22). In the publication analysis, co-citation network is an effective way to evaluate the relationships between the topics of two publications as co-citation means the frequency of which the two publications are cited at the same time (23). In the keyword analysis, co-occurrence network measures the correlation of keywords co-occurred in the same publication (24).

## 3 Results

### 3.1 Overview

**Figure 1:**
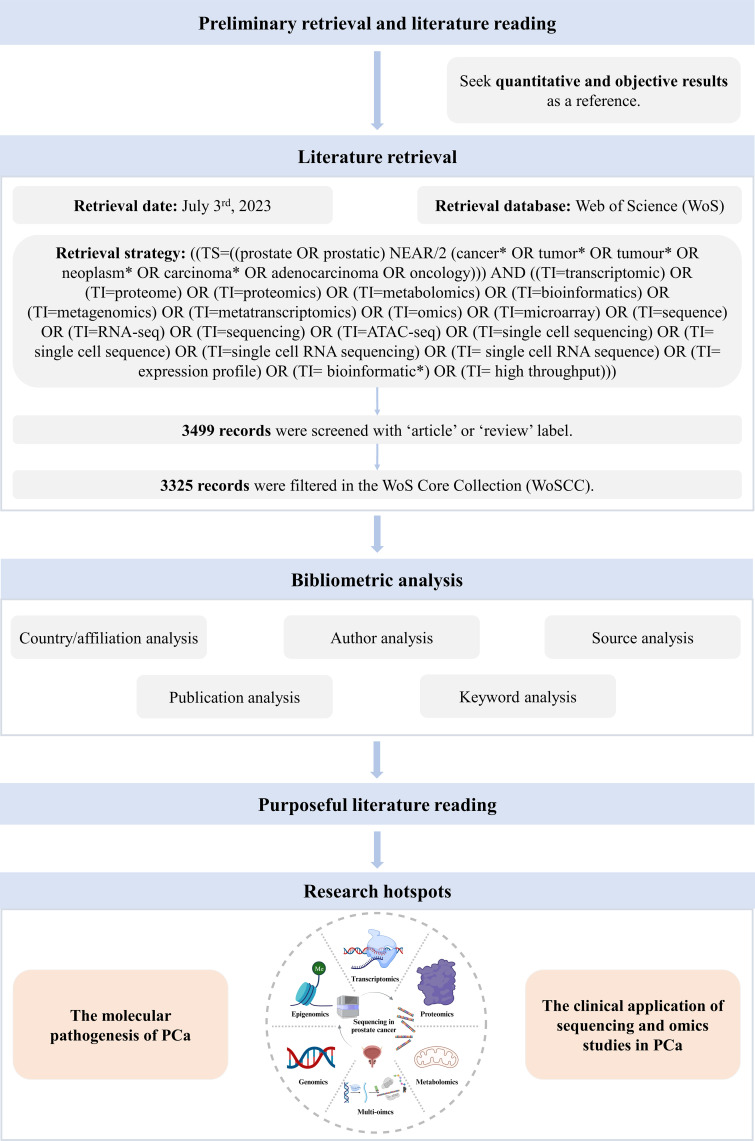
Analysis flow chart.

**Figure 2:**
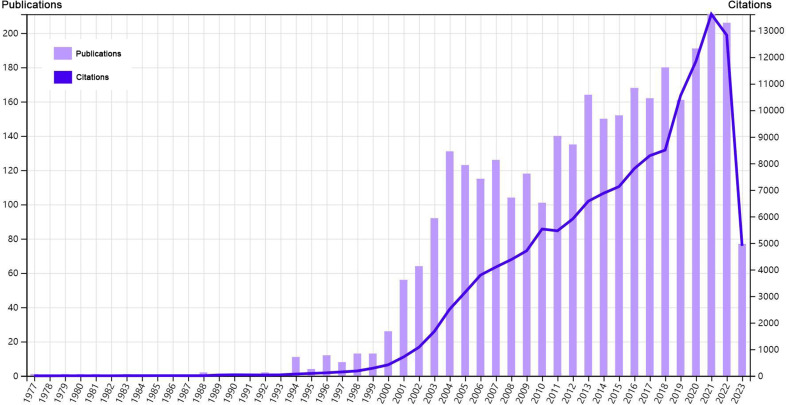
Overview of the bibliometric analysis.

Our analysis flow chart was shown in **Figure 1**. Till July 3^rd^, 2023, a total of 3225 publications were retrieved from 957 journals, books, etc. in WoS (core collection). The publication average year was 10.1, accompanied by an annual growth rate of 10.28%. The total references were 110700, and average citations per publications were 44.47. Especially, since 2001, both the publications and citations had shown an obvious increase. An explosive growth of publications and citations was seen in 2021 with a peak in **Figure 2**. As for the authors, a total of 18371 authors contributed to these publications, and 70 of them were authors of single-authored publications. The percentage of international co-authorships was 25.67%. In the publications, there were 7202 keywords plus (ID) and 5620 author’s keywords (DE) in all.

### 3.2 Countries and affiliations

**Figure 3:**
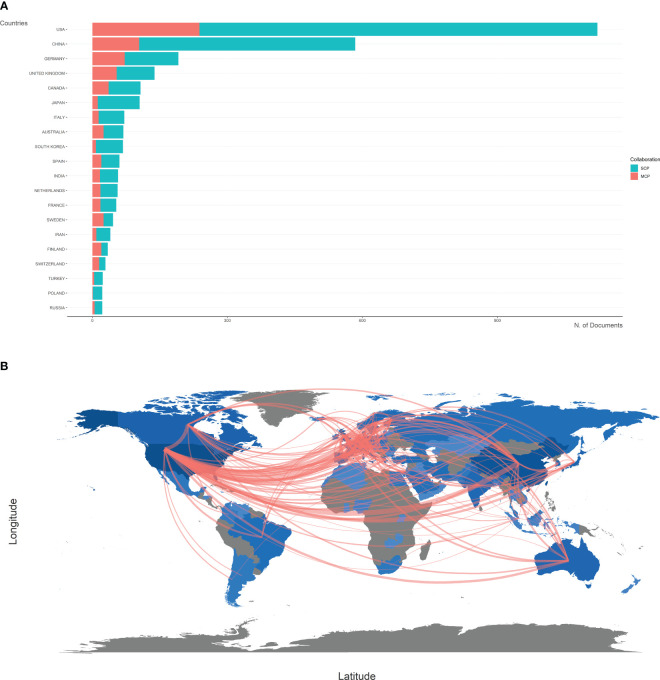
Country analysis. **(A)** The productions of top 20 countries in the field of sequencing and omics studies in prostate cancer were listed, with single-country publications and multiple-country publications shown separately. **(B)** The world map presented the productions of different countries and their collaboration with each other. SCP, single-country publication; MCP, multiple-country publication.

The top 20 most productive countries were listed in **Table 1**. The USA and China were the most productive countries in the world, but the majority of publications were restricted in single-country collaboration (**Figure 3A**). The USA, Europe, and China were three research centers of sequencing and omics studies in PCa in the world, and had close collaborations with each other (**Figure 3B**).

**Table 1:** The top 20 most relevant countries in the field of sequencing and omics studies in prostate cancer.

Rank	Country	Articles	SCPs	MCPs	MCP_Ratio	TCs	AACs
1	USA	1122	884	238	0.212	69126	61.60
2	CHINA	584	480	104	0.178	16446	28.20
3	GERMANY	191	119	72	0.377	6827	35.70
4	UNITED KINGDOM	138	84	54	0.391	6125	44.40
5	CANADA	107	71	36	0.336	4835	45.20
6	JAPAN	105	93	12	0.114	3288	31.30
7	ITALY	71	57	14	0.197	1733	24.40
8	AUSTRALIA	69	44	25	0.362	7219	104.60
9	SOUTH KOREA	68	60	8	0.118	1976	29.10
10	SPAIN	60	40	20	0.333	1683	28.00
11	INDIA	57	40	17	0.298	1675	29.40
12	NETHERLANDS	56	38	18	0.321	2087	37.30
13	FRANCE	53	35	18	0.34	1683	31.80
14	SWEDEN	46	21	25	0.543	1654	36.00
15	IRAN	40	31	9	0.225	405	10.10
16	FINLAND	34	14	20	0.588	2320	68.20
17	SWITZERLAND	29	14	15	0.517	2624	90.50
18	TURKEY	23	19	4	0.174	494	21.50
19	POLAND	22	21	1	0.045	285	13.00
20	RUSSIA	22	17	5	0.227	303	13.80

SCPs, single-country publications; MCPs, multiple-country publications; MCP_Ratio = MCPs/Articles; TCs, total citations; AACs, average article citations; AACs = TCs/Articles.

As for the corresponding authors’ affiliations, HARVARD UNIVERSITY (publications = 290), UNIVERSITY OF MICHIGAN (publications = 267), UNIVERSITY OF CALIFORNIA SYSTEM (publications = 240), NIH NATIONAL CANCER INSTITUTE (NCI) (publications = 213), and UNIVERSITY OF MICHIGAN SYSTEM (publications = 209) were the top five affiliations based on the number of publications (**Supplementary Figure S1A**). The above top five affiliations had close collaborations with each other (cluster red, cluster green, and cluster purple) (**Supplementary Figure S1B**).

### 3.3 Authors

**Figure 4:**
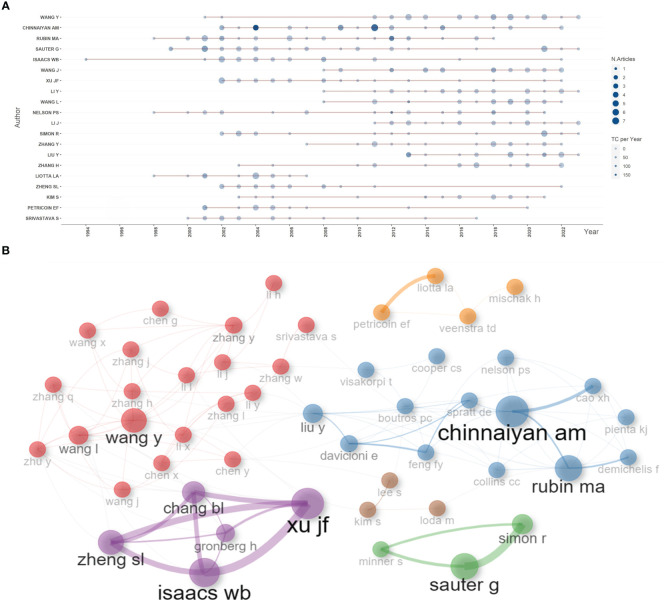
Author analysis. **(A)** The dynamic plot displayed the change of publications and total citations over time in the field of sequencing and omics studies in prostate cancer. **(B)** The collaboration network displayed the relationships among different authors in this field. TC, total citation.

Based on the number of publications, the top 20 most relevant authors were listed in **Table 2** and **Supplementary Figure S2A**. Their scientific productions over time and the total citations per year were shown in **Figure 4A**. WANG Y (publications = 37), CHINNAIYAN AM (publications = 35), RUBIN MA (publications = 32), and SAUTER G (publications = 32) far surpassed the left authors in the aspect of production. Authors’ impact was evaluated by local citations, total citations, H index, G index, and M index. CHINNAIYAN AM (local citations = 330, total citations = 8222, H index = 28, G index = 35) was regarded as the most influential author in the field of sequencing and omics studies in PCa (**Supplementary Figures S2B–D**). All the authors could be divided into several groups, and authors inside each group had close collaboration with each other (**Figure 4B**). In the field of sequencing and omics studies in PCa, the distribution of authors roughly accorded with Lotka’s Law (**Supplementary Figure S2E**).

**Table 2:** The top 20 most relevant authors in the field of sequencing and omics studies in prostate cancer.

Rank	Authors	Articles	LCs	TCs	AACs	H-index	G-index	M-index
1	WANG Y	37	48	906	24.49	18	29	0.783
2	CHINNAIYAN AM	35	330	8222	234.91	28	35	1.273
3	RUBIN MA	32	409	4081	127.53	26	32	1.000
4	SAUTER G	32	263	3425	107.03	21	32	0.840
5	ISAACS WB	24	119	2678	111.58	18	24	0.600
6	WANG J	24	105	1509	62.88	14	24	0.875
7	XU JF	23	60	1660	72.17	17	23	0.773
8	LI Y	22	7	475	21.59	10	21	0.625
9	WANG L	22	19	1379	62.68	11	22	0.688
10	NELSON PS	21	109	2183	103.95	15	21	0.577
11	LI J	20	37	547	27.35	10	20	0.769
12	SIMON R	20	50	944	47.20	15	20	0.682
13	ZHANG Y	20	20	257	12.85	11	15	0.647
14	LIU Y	19	23	1068	56.21	11	19	1.000
15	ZHANG H	18	29	588	32.67	13	18	0.619
16	LIOTTA LA	16	121	1760	110.00	15	16	.577
17	ZHENG SL	16	32	1337	83.56	13	16	0.591
18	KIM S	15	53	1111	74.07	9	15	0.429
19	PETRICOIN EF	15	119	1548	103.20	13	15	0.565
20	SRIVASTAVA S	15	61	936	62.40	13	15	0.542

LCs, local citations; TCs, total citations; AACs, average article citations; AACs = TCs/Articles.

### 3.4 Sources

Based on the number of publications, the top 20 most relevant sources were listed in **Table 3**. PLOS ONE ranked first, publishing 105 literatures, followed by PROSTATE, CANCER RESEARCH, and JOURNAL OF PROTEOME RESEARCH, with publications of 66, 56, 53, separately (**Supplementary Figure S3A**). Sources’ impact was evaluated by local citations, total citations, H index, G index, and M index. CANCER RESEARCH was regarded as one of the most influential sources in the field of sequencing and omics studies in PCa (local citations = 6378, total citations = 7983, H index = 44, G index = 56) (**Supplementary Figures S3B-D**). According to Bradford Law, the top 36 relevant journals were regarded as core sources in the field of sequencing and omics studies in PCa (**Supplementary Figure S3E**).

**Table 3:** The top 20 most relevant sources in the field of sequencing and omics studies in prostate cancer.

Rank	Sources	Articles	LCs	TCs	ACCs	H-index	G-index	M-index
1	PLOS ONE	105	2442	4224	40.23	35	62	2.188
2	PROSTATE	66	1797	1852	28.06	24	41	0.774
3	CANCER RESEARCH	56	6378	7983	142.55	44	56	0.936
4	JOURNAL OF PROTEOME RESEARCH	53	1586	1606	30.30	24	39	1.200
5	SCIENTIFIC REPORTS	45	803	1184	26.31	19	34	2.111
6	PROTEOMICS	41	1700	2219	54.12	22	41	0.957
7	BIOINFORMATICS	40	2200	2721	68.03	28	40	1.273
8	ONCOTARGET	39	1067	948	24.31	18	29	1.800
9	BMC BIOINFORMATICS	38	791	1221	32.13	18	34	0.900
10	BMC GENOMICS	34	399	2105	61.91	19	34	0.950
11	CANCERS	29	315	190	6.55	9	12	1.800
12	INTERNATIONAL JOURNAL OF MOLECULAR SCIENCES	29	459	266	9.17	10	15	0.833
13	INTERNATIONAL JOURNAL OF ONCOLOGY	29	496	1103	38.03	17	29	0.567
14	INTERNATIONAL JOURNAL OF CANCER	26	1515	1260	48.46	17	26	0.586
15	ONCOGENE	26	2030	2766	106.38	22	26	0.786
16	CLINICAL CANCER RESEARCH	25	2700	1210	48.40	20	25	0.690
17	EXPERT REVIEW OF PROTEOMICS	25	199	548	21.92	14	23	0.700
18	FRONTIERS IN ONCOLOGY	24	309	118	4.92	6	10	1.200
19	MOLECULAR & CELLULAR PROTEOMICS	24	1411	1777	74.04	20	24	0.909
20	NUCLEIC ACIDS RESEARCH	24	2892	7102	295.92	14	24	0.519

LCs, local citations; TCs, total citations; AACs, average article citations; AACs = TCs/Articles.

### 3.5 Publications

**Figure 5:**
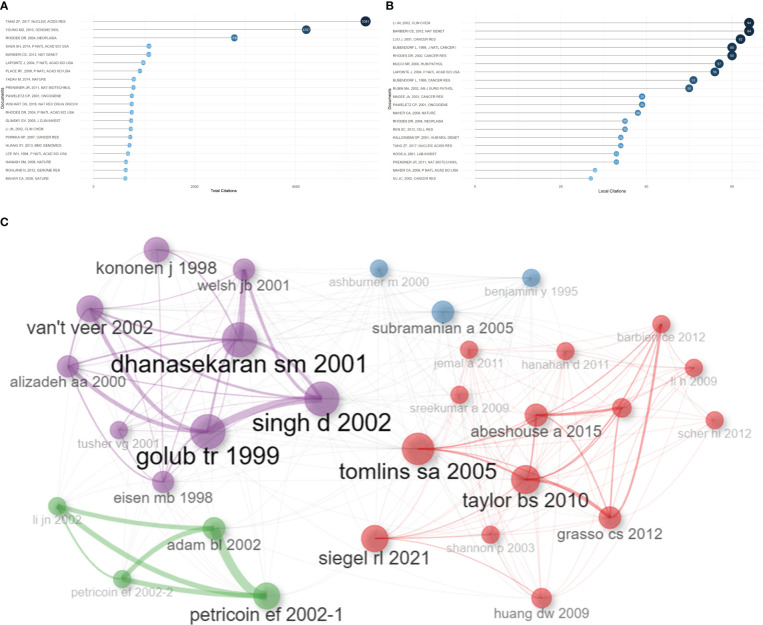
Publication analysis. **(A)** The top 20 most total cited publications in the field of sequencing and omics studies in prostate cancer were listed. **(B)** The top 20 most local cited publications were listed. **(C)** The co-cited publications were distributed in the same cluster in the co-citation network.

Based on total citations, the top 20 most total cited publications were listed in **Table 4**. ‘TANG ZF, 2017, NUCLEIC ACIDS RES’ (total citations = 5381), ‘YOUNG MD, 2010, GENOME BIOL’ (total citations = 4207), and ‘RHODES DR, 2004, NEOPLASIA’ (total citations = 2786) had the largest total impact (**Figure 5A**). Based on local citations, the top 20 most local cited publications were listed in **Figure 5B**. ‘LI JN, 2002, CLIN CHEM’ (local citations = 64), ‘BARBIERI CE, 2012, NAT GENET’ (local citations = 64), and ‘LUO J, 2001, CANCER RES’ (local citations = 62) had the largest local impact. In the co-citation network, the co-cited publications were distributed in the same cluster, thus different clusters probably represented different research hotspots in the field of sequencing and omics studies in PCa (**Figure 5C**).

**Table 4:** The top 20 most total cited publications in the field of sequencing and omics studies in prostate cancer.

Rank	Publication	LCs	TCs	LCs/TCs Ratio (%)	TCs Per Year	Raw data	Methodology	Main discovery
1	GEPIA: a web server for cancer and normal gene expression profiling and interactive analyses	34	5381	0.63	768.71	Public databases	Bioinformatic analysis	Development of a web server for interactive and customizable functions
2	Gene ontology analysis for RNA-seq: accounting for selection bias	7	4207	0.17	300.50	Self-test data of prostate cancer, liver, and kidney	Bioinformatic analysis	Development of a statistical methodology that enables the application of GO analysis to RNA-seq data
3	ONCOMINE: a cancer microarray database and integrated data-mining platform	35	2786	1.26	139.30	Public databases	Bioinformatic analysis	Development of a web-based data-mining platform for genome-wide expression analyses
4	rMATS: robust and flexible detection of differential alternative splicing from replicate RNA-Seq data	3	1097	0.27	109.70	Public databases and self-test data	Bioinformatic analysis, RT-qPCR	Development of a new statistical model and computer program to detect differential alternative splicing from replicate RNA-Seq data
5	Exome sequencing identifies recurrent SPOP, FOXA1 and MED12 mutations in prostate cancer	64	1094	5.85	91.17	Self-test data of prostate cancer	Bioinformatic analysis, RT-qPCR, immunohistochemistry, WST-1 cell proliferation experiment, Transwell experiment	Identification of new recurrent mutations in prostate cancer
6	Gene expression profiling identifies clinically relevant subtypes of prostate cancer	56	985	5.69	49.25	Self-test data of prostate cancer	Bioinformatic analysis, immunohistochemistry	Identification of clinically relevant subtypes of prostate cancer
7	MicroRNA-373 induces expression of genes with complementary promoter sequences	2	917	0.22	57.31	Self-test data of prostate cancer	RT-qPCR, ChIP analysis	Identification of a new miRNA targeting promoter sequences
8	Predicting immunogenic tumor mutations by combining mass spectrometry and exome sequencing	0	799	0.00	79.90	Self-test data of prostate cancer and colon cancer	Bioinformatic analysis, flow cytometry	Prediction of immunogenic tumor mutations
9	Transcriptome sequencing across a prostate cancer cohort identifies PCAT-1, an unannotated lincRNA implicated in disease progression	33	791	4.17	60.85	Self-test data of prostate cancer	Bioinformatic analysis, qPCR, RT-PCR, western blot, ChIP analysis, *in vitro* translational assays	Identification of a new lincRNA
10	Reverse phase protein microarrays which capture disease progression show activation of pro-survival pathways at the cancer invasion front	39	757	5.15	32.91	Self-test data of prostate cancer	Bioinformatic analysis, western blot	Identification of the state of pro-survival checkpoint proteins in the dynamic development of prostate cancer
11	Emerging applications of metabolomics in drug discovery and precision medicine	8	756	1.06	94.50	/	/	Review
12	Large-scale meta-analysis of cancer microarray data identifies common transcriptional profiles of neoplastic transformation and progression	20	755	2.65	37.75	Public databases	Bioinformatic analysis	Identification of common transcriptional profiles of neoplastic progression
13	Microarray analysis identifies a death-from-cancer signature predicting therapy failure in patients with multiple types of cancer	9	744	1.21	39.16	Public databases and self-test data	Bioinformatic analysis, anoikis assay, apoptosis assay, flow cytometry, RT-PCR, RT-qPCR	Identification of a stem cell-like expression profile in patients with multiple types of cancer
14	Proteomics and bioinformatics approaches for identification of serum biomarkers to detect breast cancer	64	738	8.67	33.55	Self-test data of breast cancer	Bioinformatic analysis	Identification of potential serum biomarkers of breast cancer
15	MicroRNA expression profiling in prostate cancer	20	727	2.75	42.76	Self-test data of prostate cancer	Bioinformatic analysis, dot blot hybridization, RT-qPCR,	Identification of the miRNA expression profiles in prostate cancer
16	Characterization of human plasma-derived exosomal RNAs by deep sequencing	3	712	0.42	64.73	Self-test data of plasma	Bioinformatic analysis, qPCR	Exploration of human plasma-derived exosomal RNAs
17	Cytidine methylation of regulatory sequences near the pi-class glutathione S-transferase gene accompanies human prostatic carcinogenesis	14	682	2.05	22.73	Self-test data of prostate cancer	Immunohistochemistry, western blot, northern blot, southern blot	Identification of epigenomic alterations associated with prostate cancer
18	Mining the plasma proteome for cancer biomarkers	15	642	2.34	40.13	/	/	Review
19	Cost-effective, high-throughput DNA sequencing libraries for multiplexed target capture	1	638	0.16	53.17	Self-test data of prostate cancer	Bioinformatic analysis	Development of a novel method for sequencing
20	Transcriptome sequencing to detect gene fusions in cancer	38	629	6.04	41.93	Self-test data of prostate cancer	Bioinformatic analysis, qPCR, FISH	Discovery of new gene fusions using integrative transcriptome sequencing

LCs, local citations; TCs, total citations; TCs Per Year = TCs/(2023–Year+1); GO, gene ontology; RT-qPCR, quantitative real-time reverse transcription PCR; ChIP, chromatin immunoprecipitation; qPCR, quantitative real-time PCR; RT-PCR, reverse transcription PCR; lincRNA, long intergenic non-coding RNA; FISH, fluorescence *in situ* hybridization.

Besides, there was a disparity in quality among the included studies. Thus, we further listed the raw data, methodology, and major discoveries of the top 20 total cited publications, in order to analyze the common methods of these high-quality studies. Generally speaking, there were two main kinds of research methods in the field of sequencing and omics studies in PCa with high quality. The first one was developing a novel web platform through bioinformatic analysis of public databases. The other one was identifying new biomarkers of PCa through bioinformatic analysis together with the corresponding molecular, cell, and animal experiments based on self-test data. These results suggested that high-quality studies in this field not only required comprehensive bioinformatic analysis, but also needed experimental validation to bolster the discoveries (**Table 4**).

### 3.6 Keywords

**Figure 6:**
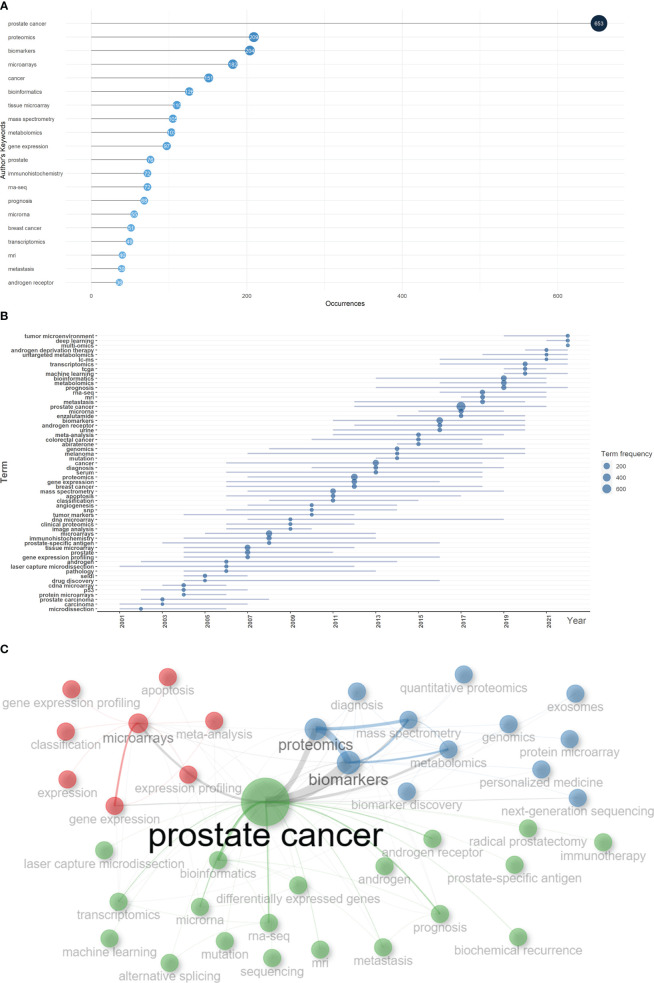
Keyword analysis. **(A)** The top 20 authors’ keywords in the field of sequencing and omics studies in prostate cancer were listed. **(B)** The dynamic plot displayed the change of keywords over time. **(C)** The keywords were distributed in different clusters in the co-occurrence map.

Based on the occurrence of authors’ keywords, the most relevant keywords were listed in **Figure 6A**, and their evolution over time was shown in **Figure 6B**. The co-occurrence network exhibited three clusters of authors’ keywords in the field of sequencing and omics studies in PCa. Cluster 1 (red) included keywords like ‘microarrays’, ‘expression’, ‘gene expression profiling’, ‘apoptosis’, etc. and might represent the molecular pathogenesis of PCa. Cluster 2 (blue) covered keywords such as ‘diagnosis’, ‘biomarkers’, ‘biomarker discovery’, ‘proteomics’, ‘metabolomics’, etc. and possibly referred to the diagnosis of PCa. Cluster 3 (green) was consisted of keywords like ‘radical prostatectomy’, ‘immunotherapy’, ‘androgen receptor’, ‘prognosis’, ‘metastasis’, ‘biochemical recurrence’, etc. and indicated the therapy and prognosis of PCa (**Figure 6C**). Above all, there were two research hotspots in the field of sequencing and omics studies in PCa: the molecular pathogenesis of PCa and the clinical application of sequencing and omics studies in PCa.

## 4 Discussion

PCa is the second most common cancer threatening the health of the elderly males worldwide (1). The major challenge in the field of PCa is the unclearness about the disease at a micro resolution, due to the limitation of technology. Nonetheless, in the past few decades, along with the rapid development of sequencing technology and the corresponding omics studies, we have witnessed a transformative evolution in the comprehension of PCa. This progress has effectively ushered researchers into the realm of the microworld of PCa, facilitating the gradual revelation of the intricate molecular biological properties characterizing the disease.

We conducted a bibliometric analysis in the field of sequencing and omics studies in PCa. Till July 3rd, 2023, a total of 3225 publications were retrieved from 957 journals, books, etc. in WoS (core collection). Since 2001, the publications had shown an obvious increase, especially for 2021. As for the most contributive countries in the world, the USA and China produced the majority of publications in this field. These two countries along with Europe harbored close collaborations with each other. WANG Y, CHINNAIYAN AM, RUBIN MA, and SAUTER G were the most productive authors in the field of sequencing and omics studies in PCa. Among them, CHINNAIYAN AM was regarded as the most influential author in the world. All the authors could be divided into several collaboration groups and maintained close relationships with each other. PROSTATE, CANCER RESEARCH, and JOURNAL OF PROTEOME RESEARCH were the top 3 journals which had the highest publications, and CANCER RESEARCH harbored the largest impact in this field. In the publications analysis, the literatures in this field were classified into different clusters in the co-citation network, possibly indicating different research hotspots. Most importantly, we listed the most frequent authors’ keywords along with their dynamic evolution over time, and ulteriorly classified these keywords into three clusters indicating different research topics. In conclusion, ‘the molecular pathogenesis of PCa’ and ‘the clinical application of sequencing and omics studies in PCa’ were two research hotspots in the field of sequencing and omics studies in PCa, and the latter one could be further classified into the susceptibility, diagnosis, therapy, and prognosis of PCa.

**Figure 7:**
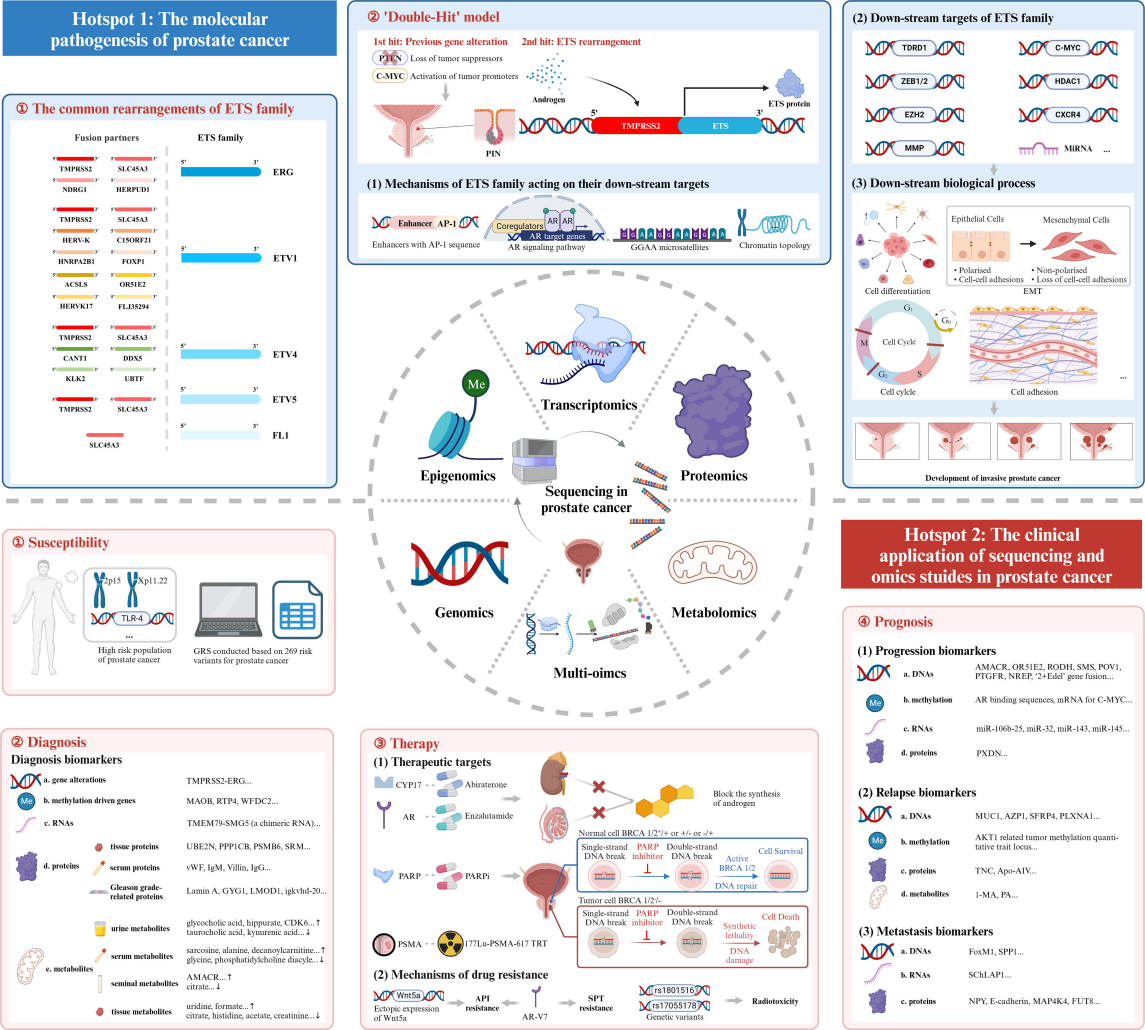
Mechanism diagram. ETS family, E26 transformation-specific family; AR, androgen receptor; EMT, epithelial-mesenchymal transition; GRS, genetic risk score; PARP, Poly ADP Ribose Polymerase; PARPi, inhibitor of PARP; PSMA, prostate-specific membrane antigen; TRT, targeted radionuclide therapy; API, androgen pathway inhibitor; AR-V7, androgen receptor splice variant 7; SPT, supraphysiological testosterone.

Based on the above bibliometric analysis, we selected the top 100 local cited publications, the top 100 total cited publications, as well as the publications in the historialgraph, co-citation network, etc. for purposeful literature reading, and summarized the achievements of different omics studies. As for genomics studies, large amounts of gene mutations in PCa were discovered, especially for the rearrangements of ETS family. Additionally, genome itself could be used as a diagnostic tool. Epigenomics studies mainly focused on epigenetic alterations occurred in PCa, like DNA methylation, and analyzed their impacts on gene expression. Transcriptomics was most frequently applied to detect DEGs and gene fusions across diverse tumor samples. Besides, some non-coding RNAs like miRNAs were also analyzed, and their special functions in PCa gradually emerged. Proteomics had advantages in detecting differentially expressed proteins as potential biomarkers for the diagnosis, prognosis of PCa. Besides, some metabolites were also identified as biomarkers in metabolomics studies. However, the profound significance of metabolomics existed in describing special metabolic phenotype of PCa. Multi-omics, which integrated genomics, epigenomics, transcriptomics, proteomics, metabolomics, etc. altogether, had unique advantages in the studies of PCa. Multi-omics studies were instrumental in constructing regulation networks in PCa, and identifying potential biomolecular mechanisms beneath the disease. Besides, multi-omics studies compensated for the limitations of single omics and harbored an advantage in accurately detecting biomarkers. Last but not least, multi-omics studies included the data of DNAs, RNAs, proteins, etc., thus providing a comprehensive model for the classification and prognosis evaluation of PCa. Above all, the research outputs of omics studies, summarized through large amounts of literature reading, could be classified into several themes encompassing the pathogenesis, susceptibility, diagnosis, therapy, and prognosis of PCa, which perfectly corresponded with the two hotspots we identified in the bibliometric analysis. In the following discussion part, we will further picture the historical trajectory of these two research hotspots in the field of sequencing and omics studies in PCa (**Figure 7**).

### 4.1 The molecular pathogenesis of prostate cancer

#### 4.1.1 The discovery of the E26 transformation-specific family rearrangements in PCa

The first research hotspot in the field of sequencing and omics studies in PCa was the molecular pathogenesis of PCa. Through bibliometric analysis and large amounts of literature reading, we found that the rearrangement of ETS family genes was regarded as the most important pathogenesis of PCa. ETS family proteins were a large family of 28 transcription factors, which had long been implicated in tumorigenesis (25). Five members of them, ERG, ETV1, ETV4, ETV5 and FL1, were seriatim identified by researchers, illustrating their oncogenic function in PCa. Tomlins et al. initially identified the rearrangements of ERG and ETV1 in 50% and 5-10% of PCa (26–28), followed by the discovery of the rearrangements of ETV4 and ETV5 (29–32). FL1, fused to SLC45A3 gene, was the fifth rearranged ETS family gene identified in PCa (33). TMPRSS2, a prostate-specific androgen reactive gene, was the most frequent gene fused to ETS family genes to form the gene rearrangements. Apart from this, other genes sharing analogous functions like SLC45A3, HERV-K_22q11.23 also took part in the rearrangements of ETS family genes. Here, we intended to elaborate the pathogenesis of TMPRSS2-ETS (+) PCa, in which the 5’ untranslated region of TMPRSS2 was fused to ETS, due to its highest occurrence in PCa (26). Other rearrangements of ETS family genes may harbor similar oncogenic mechanisms.

#### 4.1.2 The down-stream targets of ETS family genes

TMPRSS2 was activated by androgen, and launched the expression of ETS family genes. Then, the overexpressed ETS proteins combined with ETS targets and induced the down-stream signaling pathways. It was of great importance to explore the ETS target genes and the corresponding down-stream mechanisms so as to figure out the complicated oncogenic function of ETS rearrangements. Most of the studies were conducted to discover the target genes of ERG. Manuel R Teixeira et al. filtrated seven tumor-associated ERG target genes (CACNA1D, PLA1A, HLA-DMB, ATP8A2, PDE3B, TDRD1, and TMBIM1) in PCa (34). Among them, TDRD1 was the first confirmed gene that was directly regulated by ERG, and encoded the tudor domain-containing protein 1, involved in male germ cell differentiation and in the small RNAs pathway. But its special function in PCa had not been uncovered (34). Olli Kallioniemi et al. identified the up-regulation of histone deacetylase 1 (HDAC1) as the most common characteristic in PCa with TMPRSS2-ERG gene fusion (35). HDAC1 could induce the activation of urokinase-type plasminogen activator (uPA), matrix metalloprotease-1 (MMP-1) and MMP-2, but suppressed E-cadherin in order to accelerate the invasion and migration of prostate cancer cells (36–38).

Cell dedifferentiation was an important characteristic of tumorigenesis, and the differentiation degree of malignant cells usually represented the malignant grade of PCa. As for MMPs, ERG had been discovered to activate the transcription of MMP-1, resulting in loss of cell adhesion, which led to the dedifferentiation of prostate cells (39). Besides, C-MYC and polycomb group protein EZH2, a stemness-related gene, were also found to be down-stream factors of ERG influencing the dedifferentiation process (40, 41). EZH2 could also catalyze the methylation of ERG at the lysine 263 residue, and reversely enhance the oncogenic activity of ERG (42, 43).

Epithelial-mesenchymal transition (EMT) referred to the biological process of the transformation from epithelial cells to mesenchymal cells, characterized by less cell polarity and weaker cell adhesion, which played a role in oncogenesis (44). Wnt/β-catenin pathway was activated by ERG and resulted in EMT to increase the survival and invasive properties of prostate cells (45). TMPRSS2-ERG could also directly bind to Zinc finger e-box binding homeobox 1 (ZEB1) promoter and ZEB2 modulators and increase the expression of ZEB1 and ZEB2, which were key regulators in the EMT (46, 47).

Cell adhesion might affect the migration of cancer cells, and lead to the progression of cancers. Chemokine receptor type 4 (CXCR4) was activated by TMPRSS2-ERG. Combined with its ligand CXCL12, CXCR4/CXCL12 axis increased the aggressiveness of PCa and enhanced the ability of prostate cancer cells to adhere to the extracellular matrix. The axis could also indirectly accelerate the tumorigenesis via increasing the expression of MMPs (48–51).

Several other down-stream targets of ERG were also identified. MicroRNAs (MiRNAs) were another target of ERG. It had been revealed that ERG inhibited the expression of miR-200c, which down-regulated EMT-related genes and acted as a tumor suppressor (52). MiR-221, a cell cycle-related miRNA regulating the phase transition from G0 to S, showed a decreased expression in patients with TMPRSS2-ERG gene fusion (53, 54). As for cell cycle, ETS-E2F fusion oncoproteins destroyed the normal cell cycle control and took part in the tumorigenesis of PCa (55). Changmeng Cai et al. discovered that soluble guanylyl cyclase (sGC) might be a novel transcription target of TMPRSS2-ERG. The NO-cGMP pathway mediated by sGC played an important role in accelerating the proliferation of cancer cells (56).

ETV1, another important member of ETS family, also harbored unique function in PCa, even sometimes opposite to the function of ERG. Although phosphatase and tensin homolog gene (PTEN), MMPs, urokinase type plasminogen activator/urokinase type plasminogen activator receptor (uPA/uPAR) systems had been described as ETV1-related genes (33, 57), we still lacked large amounts of studies to describe the global landscape of gene expressions regulated by ETV1. We intended not to illustrate the down-stream biological process of other ETS family genes in detail, due to their relatively low occurrence of rearrangements in PCa.

#### 4.1.3 The mechanisms of oncogenic ETS family acting on their targets

Subsequent studies elaborated the complicated mechanisms of oncogenic ETS family acting on their targets. A category of enhancers combined with ETS family had been identified in PCa. These enhancers had a special ETS-binding sequence named AP-1, which was composed of dimers of JUN and FOS family transcription factors (58). ETS/AP-1 enhancer elements were found to be located near the genes associated with cell migration, cell morphogenesis, and cell development, like PLAU, VIM, etc., and might affect the expression of these genes (59). In addition, ETS family was associated with androgen receptor (AR) signaling pathways. Whole-genome ChIP-seq had shown the overlap between the ERG-binding area and AR-binding area. Thus, the overexpression of ERG may have an influence on AR-binding genes in PCa. Arul M Chinnaiyan et al. had raised a model in which the overexpression of ERG inhibited the AR-regulated differentiation but induced the dedifferentiation mediated by the H3K27 methyltransferase EZH2 (60). However, ERG showed an opposite function of enhancing AR-related pathways when PTEN, a phosphatase inhibiting the development of tumor by antagonizing the activity of phosphorylases, was lost (61). ETV1 was found to cooperate with the AR signaling pathways in LNCaP cells (hormone-dependent human PCa cell lines) and PTEN deficient mouse models of PCa. Additionally, ETV1 could also directly up-regulated genes associated with steroid hormone synthesis and androgen metabolism to increase the expression of testosterone, followed by an up-regulated transcriptional activity of AR (62, 63). Besides, Peter C. Hollenhorst et al. found that ETS family could bind an unusual regulatory sequence consisting of repeats of the sequence GGAA, called GGAA microsatellites, and activate the down-stream target genes (64). Apart from this, ETS family genes functioned as transcriptional activators or suppressors when they were combined with different molecules. CBP/p300, MED25 could combine with ETS family to perform a transcriptional activated function, while EZH2, HDAC1, HDAC2, and FOXO1 inhibited ETS-induced gene transcription (65). Last but not least, ETS family could significantly impact the topology of chromatin to influence the gene transcription, since the interaction of ETS family with many chromatin modifying enzymes like p300, HDAC1, EZH2, PRMT5, KDM4A, SETPB1, etc. (66–70).

The overall pathogenesis of TMPRSS2-ETS gene fusion in PCa could be concluded as a ‘Double-Hit’ model. Previous genetic alterations like gene mutations, inhibition of tumor suppressors and activation of tumor promoters might accelerate the progression of prostatic intraepithelial neoplasia (PIN), and the subsequent molecular biological change mediated by ETS rearrangements promoted the development of invasive PCa. The combination of the biological effects of ETS rearrangements and existed genetic alterations contributed to the tumorigenesis of PCa together (28).

### 4.2 The clinical application of sequencing and omics studies in prostate cancer

#### 4.2.1 Susceptibility

The application of sequencing and omics studies in PCa had revealed certain population that were more susceptible to PCa. In a Genome Wide Association Study (GWAS) in 2008, common sequence variants on 2p15 and Xp11.22 were found to be associated with susceptibility to PCa (71). Another study revealed toll-like receptor 4 (TLR-4), a candidate inflammatory gene, was associated to high risk of PCa when its 3’-untranslated region suffered mutations (72). Through a large multiancestry GWAS meta-analysis, 86 novel risk variants associated with the susceptibility to PCa were identified. Integrated with the known risk loci for prostate cancer, ‘genetic risk score (GRS)’ was conducted on the basis of 269 risk variants for PCa. GRS could effectively stratify the risk of PCa across populations, and had been verified in two independent cohorts of men of European and African ancestry (73).

#### 4.2.2 Diagnosis

The diagnosis of PCa was currently based on the microscopic evaluation of prostate tissue obtained via needle biopsy. A pathologist examined these tissues and reported the Gleason grade of the predominant histological pattern and the secondary histological pattern. Then, clinicians classified PCa into low-, intermediate-, and high-risk groups according to the sum of Gleason patterns, PSA, and clinical stage. However, the biomarker of PSA harbored a certain probability of false positives and false negatives, and the biopsy was an invasive procedure that may induce discomfort and pain in patients (74). We urgently demanded more specific and sensitive biomarkers to improve the diagnosis effectiveness of PCa.

Through the application of sequencing and omics studies to PCa, a series of genetic alterations had been discovered, which was significant to the diagnosis of PCa. Since the rearrangements of ETS family was the most important pathogenesis in PCa, these genetic alternations might serve as potential diagnosis markers of PCa. Mao X et al. and Arul M Chinnaiyan et al. had detected the gene fusion of TMPRSS2-ERG in circulating tumor cells and urine sediments from PCa patients, respectively (75, 76). And the combined detection of TMPRSS2-ERG and PCA3 significantly improved the sensitivity of diagnosis (77). However, TMPRSS2-ERG, as a diagnosis biomarker, had obvious limitations due to the existence of ETS gene fusion negative PCa (28). Besides, the epigenetic modifications could also perform as diagnosis biomarkers of PCa. Xue−Yi Xue et al. demonstrated key DNA methylation-driven genes like MAOB and RTP4, as novel biomarkers for the accurate prognosis of PCa (78). In 2011, Arul M. Chinnaiyan et al. painted a DNA methylation profile of PCa. CpG islands (CGI) referred to a certain area of DNA that contained large amounts of connected cytosine and guanine. CGI usually located in the transcription regulation domain, and remained non-methylated. However, in PCa, Arul M. Chinnaiyan et al. found that the methylation level was significantly up-regulated, especially for WFDC2. The promoter of WFDC2 showed recurrent methylation in PCa, but remained non-methylated in normal tissues, which might harbor diagnostic value in PCa (79). TMEM79-SMG5, one chimeric RNA, was another potential diagnostic biomarker of PCa. In comparison with TMPRSS2-ERG, which could be found in about 50% of the PCa patients, TMEM79-SMG5 was detected by RT-PCR in approximately 90% of PCa samples tested (80). Proteins were the most common biomarkers for the diagnosis of PCa. A number of differentially expressed proteins were observed in PCa tissues, like UBE2N, PPP1CB, PSMB6, metalloproteinase inhibitor-1, PCa-24, SRM, NOLC1, and PTGIS (81–84). These proteins might offer valuable insights for the diagnosis of PCa. Besides, Brian B Haab et al. identified five serum biomarkers (von Willebrand Factor, immunoglobulinM, Alpha1-antichymotrypsin, Villin, and immunoglobulinG) exhibiting significant differential expression levels between PCa samples and control samples, which may also serve as potential diagnosis biomarkers (85). Notably, several proteins were found to be in accordance to the Gleason grade of PCa. Lukas Alfons Huber et al. discovered that the expression level of Lamin A corresponded with the Gleason grade, qualifying itself as a potential biomarker for tumor differentiation and prognosis (86). Likewise, in 2019, GYG1, LMOD1, igkvhd -20, and RNASET2 were also identified as four proteins correlated with the Gleason grade of PCa (87). Metabolites well reflected the physiological process in an individual. Metabolome, located in the downstream of central dogma, was more directly associated to the phenotype and harbored more direct clinical significance, compared to genome, transcriptome, etc. The study of metabolomics provided a useful way to identify potential biomarkers in biofluid, which may offer non-invasive diagnosis indexes for PCa. The most reported biomarkers in PCa situated in urine, serum, and seminal fluid. In urine, several metabolites exhibited upregulation, including glycocholic acid, hippurate, 7-methylguanine, FABP5, BCAAs, AMBP, CDK6, etc., while taurocholic acid, kynurenic acid, glucoronate, glycine, etc. showed obvious down-regulation (81, 88–94). In serum samples, an elevated expression was observed for sarcosine, alanine, pyruvate, decanoylcarnitine, etc., but glycine, citrate, phosphatidylcholine diacyl exhibited the opposite trend (95–97). Concerning seminal fluid, citrate was down-regulated in 2 reported studies, but AMACR was up-regulated (98–100). However, the metabolomic biomarkers identified in tissues may be more significantly changed than those in biofluid. Citrate, histidine, acetate, creatinine, valine, glycine, lysine, leucine, glutamine, and choline were decreased in PCa tissues, however, uridine and formate were oppositely increased (101, 102). The number of biomarkers that had been identified was so huge to list, but hardly any of them were applied to clinical practice. Multi-omics studies might provide a novel method of diagnosis based on the integrated data. In 2018, epigenomics, transcriptomics, proteomics, and metabolomics studies were applied to a set of patients receiving radical prostatectomy. The prediction model merging the data of DNA methylation, transcripts, proteins, and glycosylation biomarkers successfully increased prognosis predicting accuracy of PCa (103).

#### 4.2.3 Therapy

The application of sequencing and omics studies in PCa helped us to discover the novel potential therapeutic targets of PCa. A series of studies had shown that the increased copy numbers and the point mutation of AR were the most frequent genetic alterations in castration-resistant prostate cancer (CRPC) (104–106). The complicated rearrangements of AR included highly recurrent tandem duplications concerned an up-stream enhancer of AR (107). More recent studies had illustrated the heterogeneity of AR-activity in primary PCa as well. The subtype of primary PCa with low AR-activity harbored similar biological characteristics to mCRPC, and was more likely to develop the resistance to ADT, docetaxel, etc. (108). Since the AR-related pathways were of great importance in PCa, several target drugs had been developed. Abiraterone, which targeted CYP17, the key enzyme in the synthesis of androgen, and enzalutamide, the second-generation AR inhibitors had been widely applied in the therapy of CRPC (109). DNA damage repair (DDR) deficiency was another frequent genetic alteration in PCa. It had been discovered that the inactivation of core homologous recombination (HR) and HR-associated genes, such as BRCA1, BRCA2, CHEK2, PALB2, CDK12, CHD1, ATM, RAD54L, RAD51B, or PTEN, occurred in a considerable proportion of localized PCa, even more frequently in metastatic PCa (110). This particular subtype of PCa was sensitive to PARPis via the mechanism of synthetic lethality, as PARPi blocked the other DDR pathway, base excision repair, as well (111). Several PARPi including olaparib, rucaparib had been studied in phase 3 trial to treat PCa (112, 113). Last but not least, prostate-specific membrane antigen (PSMA), a type II transmembrane glycoprotein, was fairly lowly expressed in normal prostate tissues, but showed a massive expression in PCa tissues, offering another therapeutic target for PCa (114). As a result, a series of PSMA target therapies, including PSMA-targeted radionuclide therapy (PSMA-TRT), PSMA-antibody-drug conjugates (PSMA-ADC), PSMA-based chimeric antigen receptor (CAR)-T cells therapy had been developed, especially for ^177^Lu-PSMA-617 TRT (115). Several relatively less reported therapeutic targets were also revealed by the application of sequencing and omics studies in PCa. Felix Y Feng et al. conducted a systematic study of mCRPC integrating whole-genome, whole-methylome, and whole-transcriptome sequencing. A novel epigenetic CpG Methylator Phenotype (CMP) subtype of mCRPC was identified, with characteristics of hyper-methylation both within and outside of CpG islands, shelves, and shores. This subtype harbored potential therapeutic significance as methylation inhibitors were FDA-approved anti-tumor drugs (116). Another transcriptomics-proteomics-metabolomics combined study revealed the CDK9 (cyclin-dependent kinase 9)-inhibition responses of PCa cells. Acute metabolic stress was induced by using pan-CDK-inhibitor AT7519. AT7519 treatment caused the accumulation of acyl-carnitines, metabolic intermediates in fatty acid oxidation (FAO) produced by carnitine palmitoyltransferase (CPT) enzymes 1 and 2. Co-inhibition of CDK9 and CPT1/CPT2 was lethal to PCa cells (117).

Apart from the potential of discovering therapeutic targets, the application of sequencing and omics studies in PCa could also help evaluate the therapeutic effect and reveal the mechanisms of drug resistance. AR-inhibitor was one of the most effective therapies in PCa, but therapeutic effects varied among patients. Transcriptomic heterogeneity of AR activity well explained the interesting phenomenon (108). Besides, patients receiving AR-inhibitor therapy had shown an activation of atypical Wnt pathway, and the ectopic expression of Wnt5a reduced the anti-proliferation function of AR inhibitors (118). Moreover, the mutations of AR also contributed to the failure of API treatment. With the assistance of sequencing technology, androgen receptor splice variant 7 (AR-V7), characterized by a truncated ligand-binding domain (LBD), was found to be associated to the drug resistance of API, as LBD was the direct target of enzalutamide and indirect target of abiraterone (119). Supraphysiological testosterone (SPT)-based therapy to some extent improved life quality of some CRPC patients, but the resistance of SPT was still a severe problem in front of us. Through transcriptomics study, Eva Corey et al. discovered that SPT durable response was associated with the sustained suppression of E2F and AR-V7 transcriptional output and the impairment of DDR. The combination therapy of SPT and AR-V7/DDR targeted drugs may well improve the response of SPT (120). Last but not least, as for radiotherapy, in 2020, a genome-wide association meta-analysis confirmed several common genetic variants associated with the susceptibility to radiotoxicity of PCa therapy, including rs1801516, rs17055178, etc. (121).

#### 4.2.4 Prognosis

##### 4.2.4.1 Progression

Omics studies had identified plenty of prognostic predictors, associated with the progression of PCa. DEGs might contribute to the progression of PCa. Compared to normal epithelial cells, 21 up-regulated genes and 63 down-regulated genes including AMACR, OR51E2, RODH, SMS, etc. were found in PIN and PCa, which may mediate the early onset of PCa. Besides, other DEGs were also observed in the transformation from PIN to PCa, including POV1, CDKN2C, EPHA4, FASN, LAMB2, etc., which contributed to the malignant progression (122). Based on machine learning, Luis Rueda et al. identified DOCK9, PTGFR, NREP, SCARNA22, FLVCR2, CLASP1, IK2F3, and USP13 as potential biomarkers predicting the progression of PCa, especially between stage II and subsequent stages (123). Besides, a special type of gene fusion could also well predict the progression of PCa. Based on the studies of ERG rearrangements in PCa, researchers had identified a novel category of PCa, characterized by ‘2+Edel’, which was defined as duplication of the fusion of TMPRSS2 to ERG sequences together with interstitial deletion of sequences 5’ to ERG. ‘2+Edel’ was found to be correlated with aggressiveness and usually resulted in worse clinical outcomes. Notably, ‘2+Edel’ might be a more reliable prognostic marker than Gleason grade, due to its stability independent of observers or time (124). As an important component of epigenetic modification, methylation could activate or silence the expression of target genes to influence the progression of PCa. Gerhardt Attard et al. described the plasma methylation profile of PCa. The methylation of AR binding sequences was related to more aggressive clinical course (125). Moreover, methyltransferase-like 3 (METTL3) increased the expression of C-MYC via up-regulating the N6-methyladenosine (m(6)A) level of mRNA for C-MYC, and resulted in the progression of PCa (126). MiRNAs were a category of small non-coding RNAs encoded in the genomes, that cleaved messages of protein-coding genes or repressed translation (127). Located in the upstream of gene expression, miRNAs mediated the differential expression of genes, and served as progression predictors as well. Carlo M. Croce et al. discovered that the expression of miR-106b-25 (regulating E2F1 and p21/WAF1), miR-32 (regulating Bim) increased along with the progression of PCa (128). In addition, miR-143 and miR-145 combined with mRNA for myosin VI (MYO6) and regulated its expression. The binding cite mutation located in the 3’-untranslated region (UTR) of the mRNA caused the loss of inhibition of miR-143 and miR-145. As a result, the expression level of MYO6 was up-regulated, which might play a role in the development of PCa (129). Proteins also played a role in the progression of PCa. Valerie A. Odero-Marah et al. used the method of proteomics-metabolomics and identified peroxidasin (PXDN) as a protector against metabolic and oxidative stress in PCa. They firstly found the increased expression of PXDN along with the progression of PCa by immunohistochemistry. The metabolomics analysis subsequently revealed increased oxidative stress, mitochondrial dysfunction, and gluconeogenesis pathways with PXDN knockdown, and metabolic reprogramming associated with decreased nucleotide biosynthesis and increased oxidative stress was induced. Further studies verified that PXDN knockdown led to a reactive oxygen species (ROS) increase associated with decreased cell viability and increased apoptosis. Overall, PXDN down-regulated cell apoptosis by inhibiting oxidative stress, leading to the progression of PCa (130). Specifically, CRPC represented an advanced stage of PCa, usually associated with clinical progression. The special molecular biological characteristics of CRPC may well explain its worse clinical outcomes. Through single cell analysis, a certain type of endothelial (activated endothelial cells, aECs) was enriched in CRPC, harboring active communications with malignant cells, and promoting the aggressiveness of CRPC (131). Mark A Rubin et al. discovered that 44% of CRPCs harbored the genomic alterations of AR, including the AR copy number increase and the AR point mutations. Other recurrent alterations included the loss of PTEN, retinoblastoma gene (RB), the mutation of tumor protein p53 gene (TP53), phosphatidylinositol-4,5-bisphosphate 3-kinase, catalytic subunit α gene (PIK3CA), etc. (104).

##### 4.2.4.2 Relapse

Relapse was another important factor leading to poor clinical outcomes of PCa. Previous studies had identified several potential relapse predictors of PCa. Jonathan R Pollack et al. applied immunohistochemistry on tissue microarrays and identified MUC1 and AZP1 as significant predictors of the relapse of PCa, independent of tumor grade, stage, and preoperative PSA levels (132). In addition, Lars Dyrskjøt et al. found that SFRP4 was an independent predictor of the relapse after prostatectomy (133). In 2019, Paul C. Boutros et al. identified one tumor methylation quantitative trait locus related to the expression of AKT1, which could be used to predict the relapse after local therapy (134). Moreover, the increased expression of PLXNA1 independently predicted the biochemical relapse of PCa (135). Based on a multi-omics discovery platform, several serum prognostic biomarkers were identified. Two proteins-Tenascin C (TNC) and Apolipoprotein A1V (Apo-AIV), one metabolite-1-Methyladenosine (1-MA) and one phospholipid molecular species phosphatidic acid (PA) showed fairly high cumulative prediction effectiveness for the biochemical relapse of PCa, which had been confirmed in the validation set. The prediction sensitivity was ulteriorly improved when combined with pTstage and Gleason grade (136).

##### 4.2.4.3 Metastasis

Metastasis was the most common adverse prognostic event in PCa, especially for bone metastasis. Large amounts of studies had been applied to find potential predictors of metastasis. In a 2007 study, hundreds of DEGs were detected in metastatic PCa, like Forkhead Box M1 (FoxM1), Osteopontin (SPP1), etc., which covered the androgen ablation related pathways as well as other metastatic pathways such as cell adhesion, bone remodeling, and so on (137). SChLAP1, a long non-coding RNA, was identified to be the highest expressed associated with the progression of metastatic PCa. Validation in three independent cohorts confirmed the unique value of SChLAP1 in the metastasis of PCa (138). As for proteins, low expression of neuropeptide Y (NPY) was considered as a risk factor that correlated with metastasis (139), and pro-NPY was identified as a specific prognosis-related biomarker, which correlated with the increase of mortality risk (140). M L Day et al. illustrated that E-cadherin was transiently down-regulated in localized PCa, but showed a fairly high expression level in metastatic PCa (141). Additionally, mitogen-activated protein kinase kinase kinase kinase 4 (MAP4K4) was up-regulated driven by MYC, and activated the process of metastasis (142). Interestingly, the increased expression of fucosyltranferase (FUT8) could reduce the number of extracellular vesicles (EVs) secreted by malignant cells in PCa and increase the protein abundance associated with metastasis (143).

The advancements in sequencing and omics studies had greatly accelerated the development of studies in PCa, especially for the exploration of the molecular pathogenesis of PCa and discovering novel biomarkers for clinical practice. However, in the past decade, a number of novel techniques had emerged and contributed to the studies of PCa. As mentioned above, with the evolution from bulk RNA-seq to scRNA-seq, the sequencing precision had stepped into a new era, providing researchers with a useful tool to study tumor heterogeneity and molecular mechanisms (144–146). The past decades had witnessed the prosperity of the application of scRNA-seq in PCa studies. Chang Zou et al. used the method of scRNA-seq, and identified three luminal clusters in the early stage of PCa (147). Daniel A Haber et al. applied scRNA-seq to 77 circulating tumor cells from 13 patients, and found that the activation of noncanonical Wnt signaling was involved in the antiandrogen resistance of PCa (118). In spite of this, due to the limitations of technology, scRNA-seq was not able to reveal the spatial locations of gene expressions. The occurrence of spatial RNA sequencing (spRNA-seq) compensated for this limitation and reserved the original appearance of tissues during the sequencing process (148). However, on account of the relatively lower sequencing resolutions, spRNA-seq was usually employed in combination with scRNA-seq. In 2020, Itai Yanai et al. integrated scRNA-seq and spRNA-seq together, and revealed the tissue architecture in pancreatic ductal adenocarcinoma (149). Up to now, there have been no relevant studies using the method of scRNA-seq and spRNA-seq in PCa. In fact, the integration of scRNA-seq and spRNA-seq did not really reach *in situ* sequencing at a single-cell resolution. The novel technique of spatial enhanced resolution omics-sequencing (stereo-seq) could *in situ* identify the gene expression profiles with a nanometer resolution and a centimeter panoramic view (150, 151). In the research field of tumors, stereo-seq could be a useful tool to study the natural development process of tumors, including local growth, nearby invasion, distant metastasis, and recurrence after treatment, and reveal the molecular mechanisms beneath them. Recently, Jia Fan et al. utilized the method of stereo-seq and successfully portrayed the spatiotemporal evolution of metastatic hepatocellular carcinoma (152). However, there were no relevant studies in PCa. More stereo-seq-based studies aiming at a comprehensive atlas of the progression and metastasis of PCa might be a promising direction in the future.

Although our study comprehensively summarized the research landscape of sequencing and omics studies in PCa, there still existed some limitations. First of all, as our retrieval timespan was set till July 3^rd^, 2023, publications after our retrieval were not included, due to the continuous update of the database. These excluded studies might cause possible bias to the bibliometric analysis. In addition, some latest research has not gained enough citations due to the limited publication time. However, bibliometric analysis could not recognize these situations and might classify these works into those less important studies. To overcome these limitations, we proceeded large amounts of purposeful literature reading in this field, and conducted our discussion mainly based on the literatures themselves, rather than simply on the bibliometric results.

## 5 Conclusion

In this article, through bibliometric analysis and large amounts of purposeful literature reading, we comprehensively understand the research landscape of sequencing and omics studies in PCa. The advancement of sequencing technology alongside its associated omics studies has led to a revelation of the molecular pathogenesis of PCa, especially for the rearrangements of ETS family. Beyond this, our analysis has also unearthed a suite of pivotal biomarkers that hold significant relevance for the susceptibility, diagnosis, therapy, and prognosis of PCa, and might provide new possibilities for the clinical practice of PCa. Our bibliometric study offered the knowledge constructures and research trends of sequencing and omics studies in PCa, for those who are interested in PCa studies and might inspire innovative explorations in this field.

## Data availability statement

The original contributions presented in the study are included in the article/**Supplementary Material**. Further inquiries can be directed to the corresponding author.

## Author contributions

BL: Conceptualization, Data curation, Formal analysis, Investigation, Methodology, Visualization, Writing – original draft, Writing – review & editing. YL: Conceptualization, Data curation, Formal analysis, Investigation, Methodology, Visualization, Writing – original draft, Writing – review & editing. YY: Conceptualization, Data curation, Formal analysis, Investigation, Methodology, Visualization, Writing – original draft, Writing – review & editing. TY: Conceptualization, Formal analysis, Methodology, Writing – review & editing. HZ: Conceptualization, Formal analysis, Methodology, Writing – review & editing. XY: Conceptualization, Formal analysis, Methodology, Writing – review & editing. RH: Conceptualization, Funding acquisition, Methodology, Writing – review & editing. WZ: Conceptualization, Methodology, Supervision, Writing – review & editing. XP: Conceptualization, Funding acquisition, Methodology, Supervision, Writing – review & editing. XC: Conceptualization, Funding acquisition, Methodology, Supervision, Writing – review & editing.

## Glossary

**Table:**

PCa	prostate cancer
WoS	Web of Science
PSA	prostate-specific antigen
ADT	androgen deprivation therapy
nmCRPC	non-metastatic castration resistant prostate cancer
mCRPC	metastatic castration resistant prostate cancer
API	androgen pathway inhibitor
PARP	Poly ADP Ribose Polymerase
PARPi	inhibitor of Poly ADP Ribose Polymerase
NGS	next-generation sequencing
ScRNA-seq	single-cell RNA sequencing
DEG	differentially expressed gene
MS	mass spectrometry
ID	keyword plus
DE	author’s keyword
EMT	epithelial-mesenchymal transition
ZEB	Zinc finger e-box binding homeobox
CXCR4	chemokine receptor type 4
MiRNA	microRNA
sGC	soluble guanylyl cyclase
PTEN	phosphatase and tensin homolog gene
uPA	urokinase type plasminogen activator
uPAR	urokinase type plasminogen activator receptor
AR	androgen receptor
GWAS	Genome Wide Association Study
TLR4	toll-like receptor 4
GRS	genetic risk score
CGI	CpG islands
CRPC	castration-resistant prostate cancer
DDR	DNA damage repair
HR	homologous recombination
PSMA	prostate-specific membrane antigen
PSMA-TRT	PSMA-targeted radionuclide therapy
PSMA-ADC	PSMA-antibody-drug conjugates
CAR	chimeric antigen receptor
CMP	CpG Methylator Phenotype
CDK9	cyclin-dependent kinase 9
CPT	carnitine palmitoyltransferase
AR-V7	androgen receptor splice variant 7
LBD	ligand-binding domain
SPT	supraphysiological testosterone
METTL3	methyltransferase-like 3
MYO6	myosin VI
UTR	untranslated region
PXDN	peroxidasin
ROS	reactive oxygen species
aEC	activated endothelial cell
RB	retinoblastoma gene
TP53	tumor protein p53 gene
TNC	proteins-Tenascin C
APOA-IV	apolipoprotein A-IV
1-MA	metabolite-1-Methyladenosine
PA	phosphatidic acid
FoxM1	Forkhead Box M1
SPP1	osteopontin
NPY	neuropeptide Y
MAP4K4	mitogen-activated protein kinase kinase kinase kinase 4
FUT8	fucosyltranferase 8
EV	extracellular vesicle.
