# Supplementary material for: Advances in sequencing and omics studies in prostate cancer: unveiling molecular pathogenesis and clinical applications
Source: Front Oncol. 2024 May 10;14:1355551. doi: 10.3389/fonc.2024.1355551 (PMC11116611; doi:10.3389/fonc.2024.1355551)
Supplement: Supplementary file 1 [file DataSheet_1.docx]

**Supplementary Material**

**
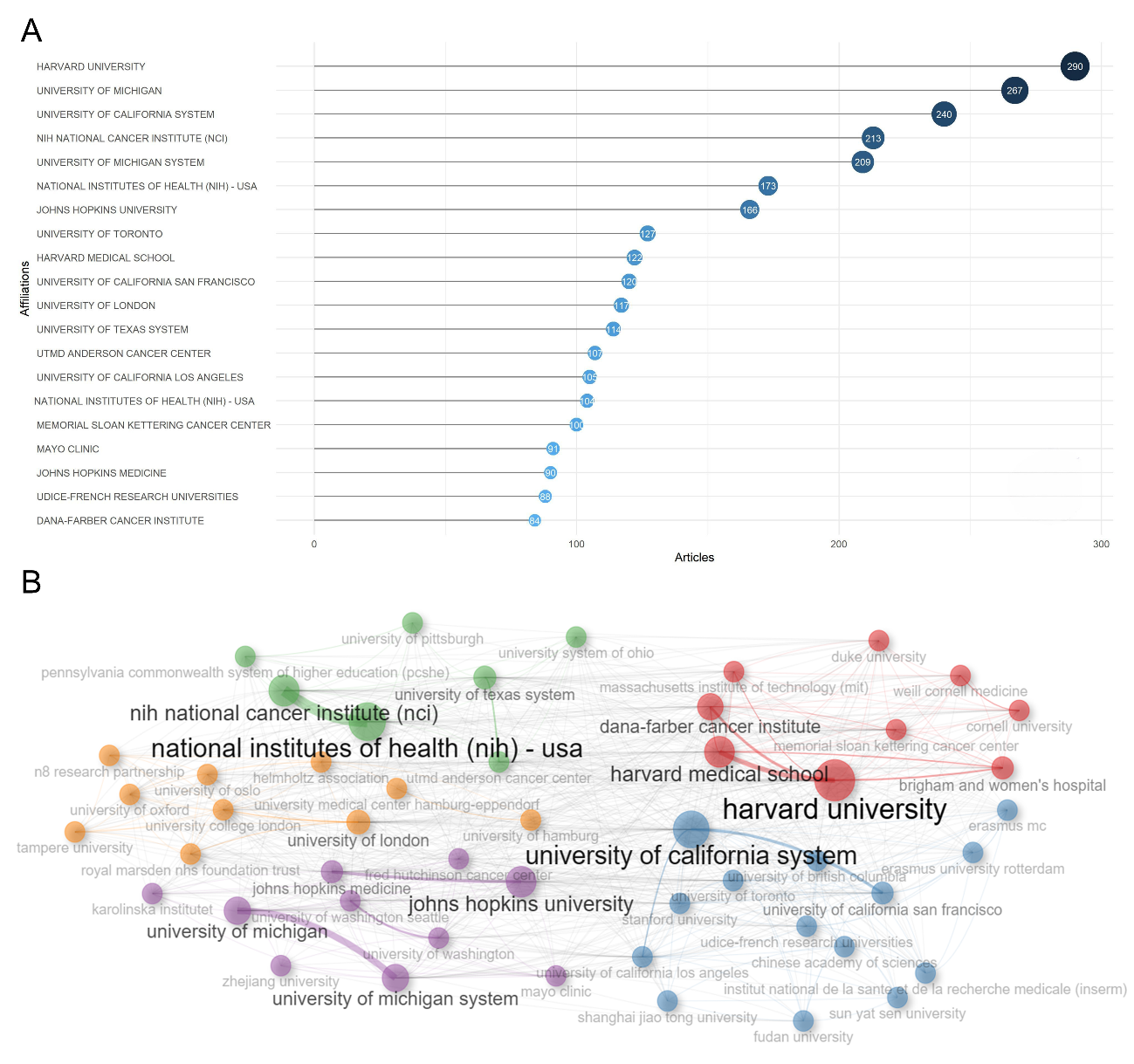
**

**FigS1.** Affiliation analysis. **(A)** The top 20 most productive affiliations in the field of sequencing and omics studies in prostate cancer were listed. **(B)** The collaboration network displayed the relationships among different affiliations in this field.


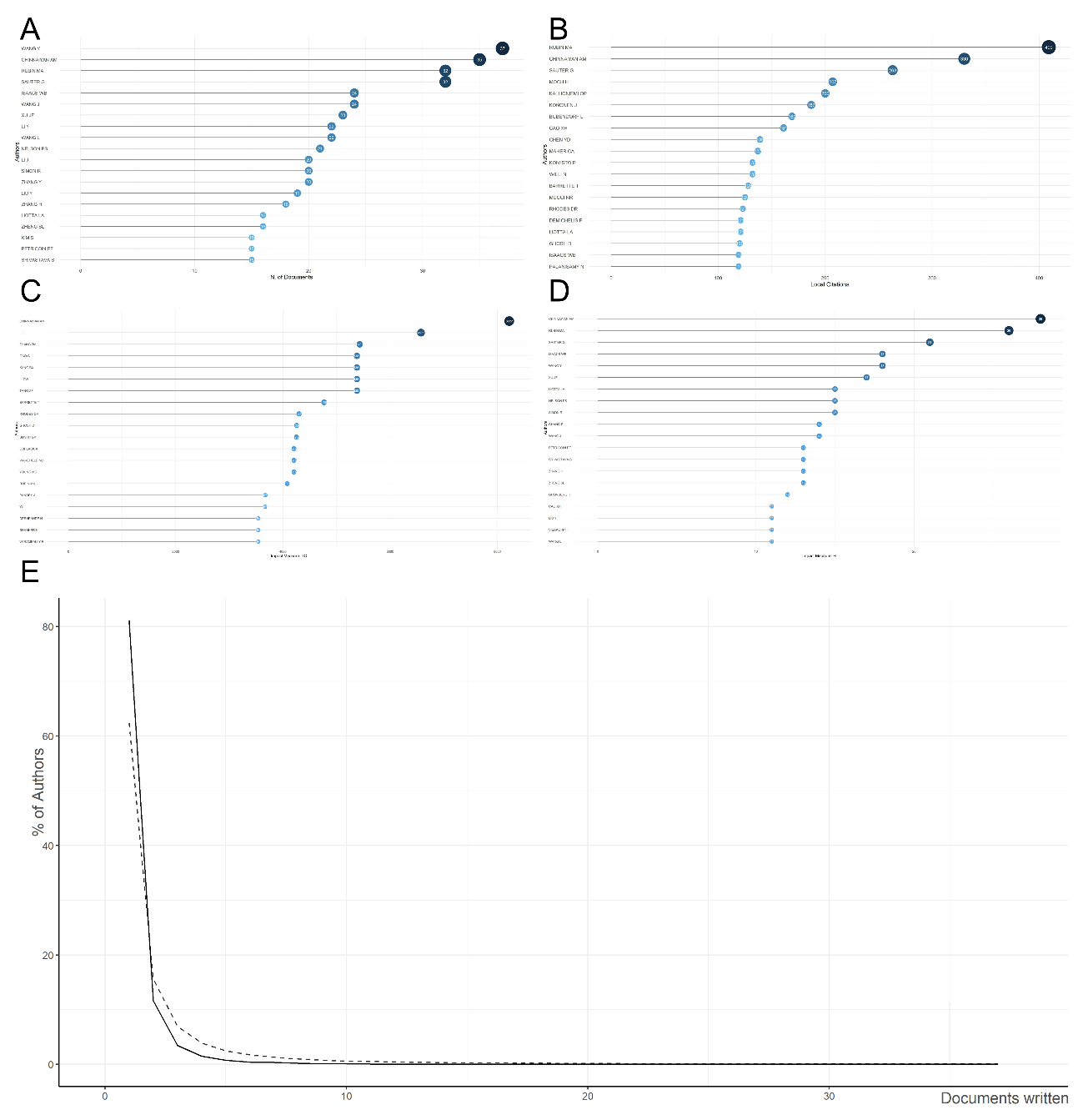


**FigS2.** Author analysis. **(A)** The top 20 most productive authors in the field of sequencing and omics studies in prostate cancer were listed. **(B)** The top 20 most local cited authors were listed. **(C)** The top 20 most total cited authors were listed. **(D)** The top 20 authors with high H index were listed. **(E)** The distribution of authors roughly accorded with Lotka’s Law. TC, total citation.


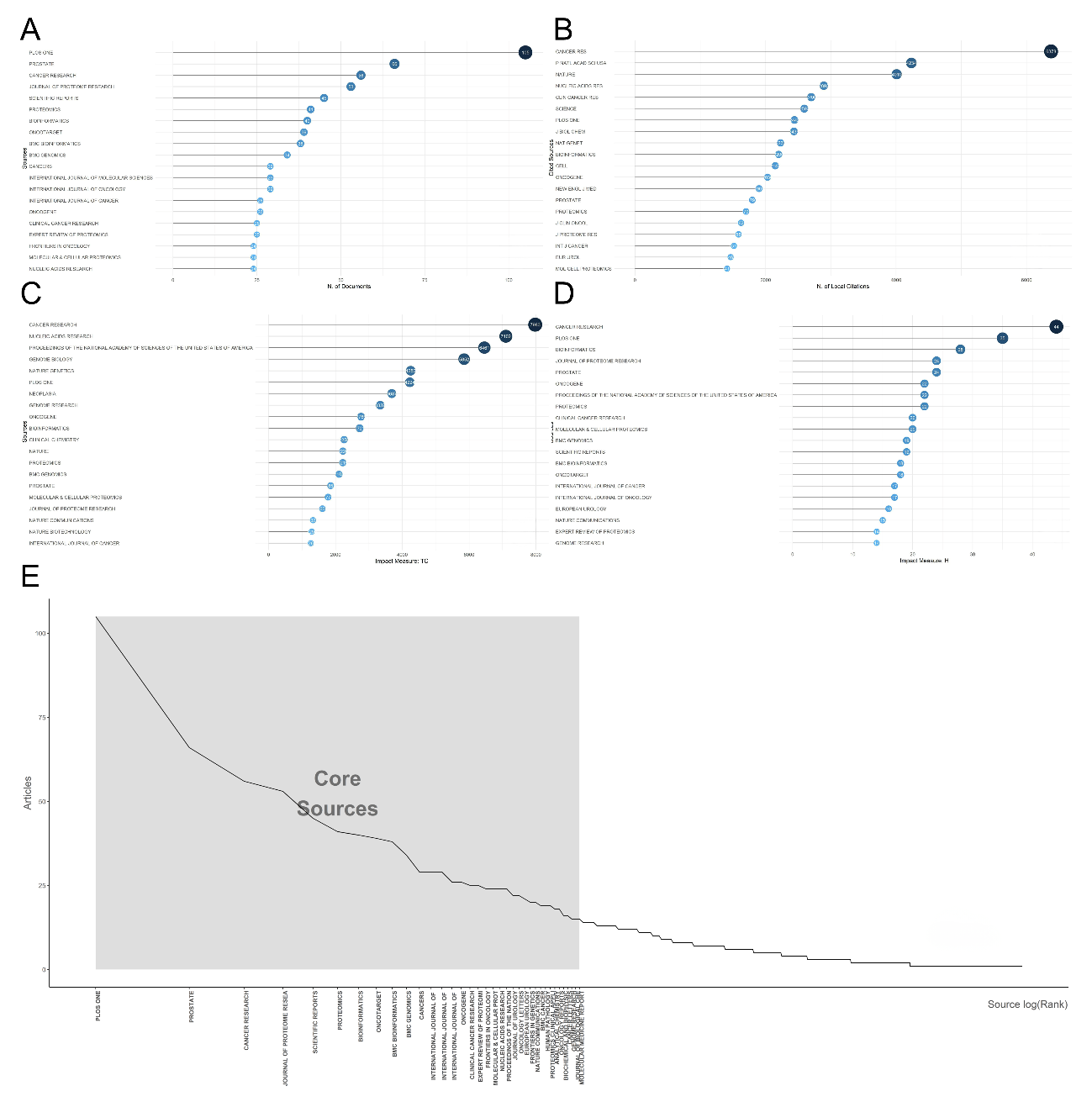


**FigS3.** Source analysis. **(A)** The top 20 most productive sources in the field of sequencing and omics studies in prostate cancer were listed. **(B)** The top 20 most local cited sources were listed. **(C)** The top 20 most total cited sources were listed. **(D)** The top 20 sources with high H index were listed. **(E)** The top 36 relevant journals were regarded as core sources in the field by Bradford Law. TC, total citation.
